# Correlates of Reported and Recorded Time Spent in Physical Activity in Working Adults: Results from the Commuting and Health in Cambridge Study

**DOI:** 10.1371/journal.pone.0042202

**Published:** 2012-07-31

**Authors:** Jenna Panter, Simon Griffin, David Ogilvie

**Affiliations:** Medical Research Council Epidemiology Unit and UKCRC Centre for Diet and Activity Research (CEDAR), Institute of Public Health, Cambridge, United Kingdom; Yale University School of Medicine, United States of America

## Abstract

**Background:**

The correlates of physical activity in adults are relatively well studied. However, many studies use self-reported (‘reported’) measures of activity and we know little about the possible differences between the correlates of reported and objective (‘recorded’) measures of physical activity. We compared the correlates of reported and recorded time spent in moderate-to-vigorous physical activity (MVPA) in a sample of working adults.

**Methods:**

In 2009, participants in the Commuting and Health in Cambridge study completed questionnaires assessing individual, socio-demographic, health and contextual characteristics. Recorded time spent in MVPA over seven days was ascertained using accelerometers and reported time spent in MVPA was assessed using the Recent Physical Activity Questionnaire (RPAQ). Correlates of MVPA were investigated using sex-specific linear regression models.

**Results:**

486 participants (70% women) provided both reported and recorded physical activity data. 89% recorded at least 30 minutes of MVPA per day. In men, none of the potential explanatory variables were associated with both reported and recorded time spent in MVPA. In women, of all the potential explanatory variables only that of having a standing or manual occupation was associated with both reported (+42 min/day; 95% CI 16.4 to 68.4, p = 0.001) and recorded (+9 min/day; 95% CI: 3.5 to 15.7, p = 0.002) time spent in MVPA.

**Discussion:**

The use of an objective measure of physical activity may influence the correlates which are observed. Researchers may wish to consider using and analysing recorded and reported measures in combination to gain a more complete view of the correlates of physical activity.

## Introduction

Given the potential health benefits of physical activity and the insufficient levels of participation in many countries, [1] promoting physical activity is a global priority. [2] In order to develop effective interventions, a clear understanding of the influences on physical activity behaviour is required.

The correlates of physical activity behaviour may at first seem relatively well studied; even ten years ago a review [3] found over 300 papers published on this topic. The authors concluded that a range of individual characteristics were associated with physical activity participation, including socio-economic status and self-efficacy. In the last 15 years researchers have increasingly recognised the potential importance of broader environmental influences on physical activity behaviour, [4] as postulated by the ecological model. [5] However, there is less consistent evidence concerning the broader environmental correlates of physical activity, including those related to the context in which behaviour is performed. [4], [6], [7] Relatively few studies have examined the associations between a range of different types of explanatory variables (for example, individual, socio-demographic, health and contextual characteristics) and objectively-measured physical activity [3].

The measurement of physical activity itself represents a significant area of methodological development, which has important implications for understanding the *correlates* of behaviour. Many authors have highlighted the importance of accurate and practical assessment of physical activity in epidemiology. [8], [9] In short, self-reported (‘reported’) measures are predominantly used to quantify levels of physical activity in large studies. This is probably because they often impose lower burden on participants, are relatively inexpensive and easy to administer en masse and can give details of the types of behaviour and the context in which behaviours are undertaken. [8], [9] However, under- or over-reporting of behaviours may occur in some population groups and this may result in biased conclusions regarding the influences on behaviour. [10] In contrast, objectively measured (‘recorded’) data from activity monitors can provide a more precise estimate of intensity, frequency and duration of physical activity and do not suffer from the recall bias of self-reported measures [8].

Although there is a wide literature on the agreement between reported and recorded measures of physical activity behaviour, [11] few studies have investigated the correlates of activity using both types of measure, [10], [12], [13] and to our knowledge only one had the primary purpose of comparing the correlates of behaviour according to the different measures. [10] Its authors found a range of correlates to be associated with both reported and recorded physical activity in a Australian sample; the direction of association was consistent across both measures for some, but not all, of these correlates. [10] Of the 16 potential explanatory variables tested in sex-stratified models, only three – lower education level and manual occupation, as well as higher mental health scores (in men) and lower alcohol consumption (in women) – were associated with both reported and recorded physical activity. Previous studies were conducted in relatively narrow population groups such as people with type 2 diabetes [12] and young adults. [10] It is important to investigate the correlates of moderate-to-vigorous activity because this may be associated with the greatest health benefits, [14] is often the focus of health recommendations [15] and may be particularly important for weight control. [16] Understanding how a variety of modifiable and non-modifiable characteristics are associated with physical activity is important as interventions to promote activity may be most effective when they target the underlying factors that influence physical activity or are tailored for different individuals. The current study aimed to compare the socio-demographic, health and contextual correlates of reported and recorded time spent in moderate-to-vigorous physical activity (MVPA) in a sample of healthy working adults in Cambridge, UK.

## Methods

### Ethics Statement

Ethical approval for the study was obtained from the Hertfordshire Research Ethics Committee and all participants gave written informed consent. Minors or children were not involved in this study.

### Study Design and Sample

These analyses use data collected as part of the Commuting and Health in Cambridge study, for which the study design, recruitment and sampling procedures have been reported elsewhere. [17], [18] Briefly, adults over the age of 16 working in Cambridge and living within a radius of approximately 30 km of Cambridge city centre were invited to take part through a predominantly workplace-based recruitment strategy. Many of the workplaces were members of the Cambridgeshire Travel for Work partnership and all were located within Cambridge, but there was heterogeneity in their geographical setting, which spanned city centre and urban fringe locations. A range of types of workplaces were approached, including local authorities, healthcare providers and retail outlets as well as institutions of higher and further education. Participants were asked to complete a questionnaire and wear an accelerometer for seven days and return both in the freepost envelope provided. Data collection occurred between May and November 2009, but the data collection periods for reported and recorded MVPA were neither synchronous nor of matching duration.

### Outcome 1: Reported Physical Activity

The Recent Physical Activity Questionnaire (RPAQ) is designed to assess home-based, occupational, recreational and transport-related physical activity in the last four weeks, from which measures of physical activity energy expenditure (PAEE) and total energy expenditure (TEE) can be estimated. A validation study of RPAQ using a small sample of adults of working age has shown the estimated PAEE to have good test-retest reliability and unusually strong criterion validity against PAEE. [19] Daily domain-specific minutes spent in MVPA were derived by summing the total time spent on relevant activities classified as ≥3 times metabolic equivalent (3 METs) according to the physical activity compendium. [20] These were then summed to derive the total reported daily time in MVPA.

### Outcome 2: Recorded Physical Activity

Free-living physical activity was assessed over one week using the ActiGraph activity monitor (GT1M, Actigraph LCC, Pensacola, US). Actigraph devices are widely used in physical activity research [21] and studies have shown that the outputs produced from these are significantly correlated with estimates of energy expenditure from studies using doubly-labelled water. [22] Participants wore the accelerometer on an elastic waistband on the right hip during waking hours, except whilst bathing and during other aquatic activities. Data were stored at 5-second intervals. A bespoke program (MAHUffe, http://www.mrc-epid.cam.ac.uk) was used for data reduction and further analyses, which removed data indicating periods of ≥10 minutes of continuous zero activity counts and any days with ≤500 minutes of recording. Participants with <3 valid days of recording were excluded from analysis. The outcome used was daily time spent in MVPA, defined as the average number of minutes per day in which >1952 counts per minute were recorded. This corresponds to the physiological intensity cutpoint of 3 METs during treadmill locomotion [23].

### Potential Explanatory Variables

Characteristics of participants (date of birth, gender, educational qualifications, possession of a driving licence, presence of long-term limiting illness or disability, type of occupation and self-reported height and weight) and their households (housing tenure, household composition, access to cars and bicycles) were assessed. Occupation type was self-reported using one of four categories (sedentary, standing, manual or heavy manual). Body mass index (BMI) was calculated by dividing weight by height squared (kg/m^2^) and participants were categorised as either ‘normal or underweight’, ‘overweight’ or ‘obese’ based on internationally recognised cut offs. [24] General health and wellbeing were assessed using the 8-item Medical Outcomes Short Form Survey (SF-8) from which physical (PCS-8) and mental (MCS-8) health summary scores were calculated and scaled using standard methods [25] and categorised into quartiles.

Each participant’s home postcode was geocoded and each location was then assigned to the respective Census Output Area (OA) and LSOA (Lower Super Output Area) using a Geographical Information System (ArcGIS 9.1). Urban-rural status and Index of Multiple Deprivation (IMD) were assessed based on the OA and derived using the classification of Bibby and Shepherd. [26] The IMD contains seven deprivation domains and IMD scores were categorised into quartiles. Data describing the quantity of greenspace were obtained from the Generalised Land Use Database [27] and assigned at LSOA level. Participants were also categorised according to the season in which their questionnaire was completed (May to August versus September to November).

### Analysis

Descriptive data were summarized using percentages and chi-squared tests (for categorical measures) and t-tests (for continuous measures). The frequency of missing data on individual, socio-economic and contextual characteristics and on physical activity was low (<0.25%). Missing responses for explanatory variables were conservatively imputed with the responses least likely to be associated with physical activity according to published literature. [3], [5], [7] Analyses were also stratified by gender because recorded time spent in MVPA differed between men and women (mean daily minutes: 59.65 versus 52.82; *p* = 0.01) and previous research had suggested that correlates of physical activity differed by gender [3].

Linear regression analyses were conducted to examine univariate associations between individual, socio-demographic, health and contextual characteristics and reported or recorded time spent in MVPA. Age and weight status (both hypothesised *a priori* to be associated with physical activity [3]) and all other variables associated with MVPA at *p*<0.25 in univariate analysis [28] were carried forward to multiple regression models. Models involving recorded time spent in MVPA were adjusted for accelerometer wear time as participants who wore the accelerometer for longer accumulated more minutes of moderate, vigorous and very vigorous activity. All analyses were undertaken in Stata version 11.

## Results

### Sample Characteristics

714 study participants were issued with questionnaires and accelerometers and 499 returned a completed questionnaire and an accelerometer that had been worn. Of these 486 (68% of those issued) provided at least 3 valid days of accelerometer data and were included in analysis. Characteristics of the main study sample (n = 1164) and those who provided both reported and recorded MVPA data (n = 486) are also shown in Table 1. Compared to the main study sample, participants included in this analysis were older (mean age 43.1 versus 41.6 years, p = 0.023), more likely to have access to a car (89.9% versus 81.4%, *p*<0.001), own their home (79.6% versus 67.4%, p<0.001), live in a rural area (44.4% versus 26.6%, *p*<0.001) and live in an area with a high proportion of greenspace (47.3% versus 34.6%, *p*<0.001), and were less likely to live in an area in the lowest deprivation quartile (16.26% versus 31.3%, *p*<0.001).

**Table 1:** Sample characteristics.

	Percentage (n)					
	Total study sample (n = 1164)			Sample for analysis (n = 486)		
	Men (n = 359)	Women (n = 786)	p	Men (n = 148)	Women (n = 338)	p
*Socio-demographic characteristics*						
Age in years						
16–30	11.2 (41)	19.7 (156)	0.002	7.4 (11)	16.6 (67)	0.021
30–40	27.5 (101)	28.5 (226)		27.0 (40)	27.5 (93)	
40–50	29.2 (107)	198 (25)		33.1 (49)	23.7 (80)	
Over 50	32.1 (118)	213 (27		32.5 (48)	32.2 (109)	
Number of adults in the household						
Single-person household	14.7 (54)	18.1 (145)	0.046	10.8 (16)	18.0 (61)	0.054
Two adults in household	65.1 (239)	57.5 (458)		68.9 (102)	58.3 (197)	
Three or more adults in household	20.1 (74	34.4 (194)		20.3 (30)	23.7 (80)	
Number of children in the household						
No children in the household	77.4 (284)	81.2 (647)	0.133	75.0 (111)	79.8 (270)	0.229
At least 1 child in the household	22.6 (83)	18.8 (150)		25.0 (37)	20.2 (68)	
Access to bicycle						
No	9.6 (35)	18.6 (147)	0.001	9.5 (14)	19.2 (65)	0.008
Yes	91.4 (330)	81.4 (644)		90.5 (133)	80.9 (272)	
Work type						
Sedentary	83.6 (306)	79.0 (629)	0.067	82.3 (121)	81.3 (274)	0.793
Standing/Manual	16.4 (60)	21.0 (167)		17.7 (26)	18.7 (63)	
Educational qualifications						
Less than degree	18.6 (68)	32.0 (253)	0.001	18.2 (27)	33.8 (112)	0.001
Degree	81.4 (298)	67.9 (536)		81.8 (121)	66.2 (224)	
Housing tenure						
Rents home	24.9 (91)	28.8 (228)	0.168	18.9 (28)	21.0 (71)	0.589
Owns home	75.1 (275)	71.2 (565)		81.1 (120)	79.0 (266)	
Car access						
No access to a car	15.8 (58)	14.7 (117)	0.618	13.5 (20)	8.6 (30)	0.096
Access to one or more cars	84.2 (309)	85.3 (680)		86.5 (128)	91.4 (309)	
Index of multiple deprivation						
Least deprived	31.0 (114)	36.8 (293)	0.205	22.3 (33)	26.6 (90)	0.363
2^nd^ quartile	25.3 (93)	25.2 (201)		34.3 (36)	24.9 (84)	
3^rd^ quartile	22.7 (83)	20.5 (163)		23.7 (35)	26.0 (88)	
Most deprived	21.0 (77)	17.5 (139)		29.7 (44)	22.5 (76)	
*Health characteristics*						
Long term illness						
No	91.3 (334)	89.0 (706)	0.245	91.2 (135)	88.1 (297)	0.316
Yes	8.7 (32)	11.0 (87)		8.8 (13)	11.9 (40)	
Weight status						
Normal weight	57.7 (207)	65.1 (512)	0.015	54.1 (78)	64.9 (217)	0.026
Overweight/Obese	43.3 (152)	38.9 (274)		45.8 (66)	35.1 (117)	
Physical health summary score						
Q1 (low)	21.7 (81)	24.5 (194)	0.475	22.3 (33)	26.4 (89)	0.252
Q2	28.9 (86)	24.9 (197)		29.7 (44)	21.4 (72)	
Q3	25.0 (86)	24.9 (197)		23.7 (35)	25.2 (85)	
Q4 (high)	24.4 (89)	25.8 (204)		24.3 (36)	27.0 (91)	
Mental health summary score						
Q1 (low)	22.3 (81)	27.4 (217)	0.014	21.6 (32)	26.7 (90)	0.109
Q2	23.6 (86)	28.8 (228)		21.0 (31)	27.9 (94)	
Q3	23.6 (86)	19.4 (154)		30.4 (45)	23.4 (79)	
Q4 (high)	30.5 (111)	24.4 (193)		27.0 (40)	22.0 (74)	
*Contextual characteristics*						
Season of data collection						
May to August	67.4 (244)	65.6 (515)	0.550	77.1 (111)	72.4 (241)	0.283
September to November	32.6 (118)	34.4 (270)		22.9 (33)	27.6 (92)	
Percentage of green space						
Q1 (lowest provision)	34.8 (128)	33.9 (270)	0.955	29.0 (43)	23.4 (79)	0.499
Q2	27.0 (99)	26.5 (211)		23.0 (34)	27.2 (92)	
Q3	21.0 (77)	21.1 (168)		22.3 (33)	24.9 (84)	
Q4 (highest provision)	17.2 (63)	18.5 (147)		25.7(38)	24.5 (83)	
Urban-rural status						
Urban	29.1 (107)	36.3 (289)	0.005	54.7 (81)	55.9 (189)	0.808
Rural	70.9 (260)	63.7 (507)		45.3 (67)	44.1 (149)	
*Physical activity*						
Mean reported daily time spent in MVPA (SD)	186.68 (124.88)	151.61 (101.56)	0.002	197.54 (112.61)	147.51 (94.88)	0.001

Quartiles of IMD defined with reference to the sample population in analysis. p values refer to a test of the difference between men and women within the respective samples. Missing responses imputed with the most conservative responses (least likely to be associated with MVPA) to maximise sample size (n = 8 cases across all variables).

Overall this sample of adults were relatively active, with over 96% of participants reporting an average of at least 30 minutes of MVPA per day over the last four weeks (which corresponds to the minimum level of physical activity recommended by the UK Chief Medical Officers [15]), whilst 89% recorded at least 30 minutes per day in MVPA. The level of agreement between the two measures was moderate (kappa  = 0.40; Table 2).

**Table 2:** Reported time spent in MVPA by recorded time spent in MVPA.

	Reported <30 mins of MVPA	Reported > = 30 mins of MVPA	Total
Recorded <30 mins of MVPA	4	48	52
Recorded > = 30 mins of MVPA	12	422	434
Total	16	470	486

Numbers shown represent the number of participants in each group.

### Correlates of Reported and Recorded Time Spent in MVPA

When examined individually, four potential explanatory variables were associated with *reported* time spent in MVPA in men and all remained significant in the multiple regression model (Table 3). Higher reported time spent in MVPA was associated with having a standing or manual occupation and with not having at least a degree level qualification. Access to a bicycle was associated with an additional 99.9 minutes of MVPA per day (95% CI 44.1 to 155.9, *p* = 0.001), and men reporting mental health in the top quartile reported an additional 72.0 minutes of MVPA per day compared with those in the lowest quartile (95% CI 26.0 to 117.9, *p* = 0.010). For men’s *recorded* time spent in MVPA, eight variables were associated in univariate analysis and two remained significant in the multiple regression model. The numbers of adults in the household and living in an area with a higher proportion of greenspace were negatively associated with recorded time spent in MVPA.

**Table 3:** Associations between individual, socio-economic and area-level characteristics and time spent in moderate and vigorous physical activity in men.

	Average daily reported time in MVPA					Average daily recorded time in MVPA					
	Univariate associations β(95%CI) p			Multiple regressionmodel β (95%CI) p		Univariate associations β(95%CI) p		Multiple regressionmodel β (95%CI) p			
*Socio-demographic characteristics*											
Age group (reference: Under 40 years)											
40–50	29.39 (−14.71, 73.50)		0.401	30.53 (−10.18, 71.24)	0.051	−2.82 (−12.02, 6.38)	0.220	3.31 (−7.42, 14.04)			0.552
Over 50 years	−19.88 (−64.22, 24.46)			−40.70 (−82.24, 0.85)		−7.33 (−16.59, 1.92)		−2.95 (−13.69, 7.80)			
Adults in the household (reference: Single person)											
Two adults	−18.99 (−78.88, 40.90)		0.531	n.i		−18.21 (−20.60, 4.17)	0.247	−**7.05 (**−**19.72, 5.62)**			**0.047**
Three adults or more	10.71 (−58.25, 79.66)					−9.58 (−23.84, 4.68)		−**11.37 (**−**25.94, 3.20)**			
Children in the household (reference: None)											
At least 1 child in the household	−0.39 (−42.78, 42.00)		0.985	n.i		−3.19 (−11.95, 5.56)	0.472	n.i			
Access to a bicycle (reference: no)											
Yes	79.80 (18.24, 141.37)		0.011	**99.98 (44.06, 155.91)**	**0.001**	−12.54 (−25.37, 0.29)	0.055	−8.56 (−22.41, 5.30)			0.224
Educational qualifications (reference: less than degree)											
Degree	−67.26 (−113.50, −21.02)		0.005	−**50.53 (**−**93.33,** −**7.73)**	**0.021**	5.46 (−4.30, 15.29)	0.270	n.i			
Work type (reference: sedentary)											
Standing/Manual	117.17 (72.80, 161.55)		0.001	**117.89 (75.47, 160.32)**	**0.001**	6.28 (−3.67, 16.25)	0.215	6.14 (−4.14, 16.42)			0.240
Housing tenure (reference: rents home)											
Owns home	−5.72 (−52.58, 41.14)		0.810	n.i		−6.59 (−16.24, 3.04)	0.178	1.37 (−9.94, 12.68)			0.811
Car access (reference: none)											
At least 1 car in the household	27.34 (−26.17, 80.85)		0.314	n.i		−11.43 (−22.38, −0.47)	0.041	−4.39 (−17.44, 8.66)			0.507
Index of multiple deprivation (reference: least deprived)											
2^nd^ quartile	−28.87 (−82.79, 25.04)		0.859	n.i		10.51 (−0.54, 21.56)	0.161	15.55 (4.17, 26.94)			0.110
3^rd^ quartile	−25.21 (−79.50, 29.07)					6.65 (−4.48, 17.78)		9.39 (−1.78, 20.55)			
Most deprived	−9.19 (−60.70, 42.33)					9.62 (−0.94, 20.18)		11.52 (0.93, 22.11)			
Average daily wear time (in minutes)	–			–		0.07 (0.03, 0.12)	0.002	**0.08 (0.03, 0.13)**			**0.002**
*Health characteristics*											
Long term illness (reference: no)											
Yes	−25.58 (−90.30, 39.14)	0.436		n.i		−3.06 (−16.47, 10.35)	0.652	n.i			
Weight status (reference: normal weight)											
Overweight/Obese	−7.79 (−45.34, 29.76)	0.683		14.57 (−18.89, 48.02)	0.391	2.80 (−4.80, 10.41)	0.467	3.28 (−4.75, 11.31)		0.421	
Physical health summary score (reference: Q1)											
Q2	9.97 (−41.78, 61.71)	0.890		n.i		0.56 (−10.08, 11.21)	0.436	n.i			
Q3	−0.57 (−55.09, 53.95)					−2.11 (−13.33, 9.11)					
Q4	8.09 (−46.06, 62.24)					5.38 (−5.77, 16.52)					
Mental health summary score (reference: Q1)											
Q2	43.23 (−12.21, 98.68)	0.066		**36.46 (**−**12.82, 85.73)**	**0.010**	−1.77 (−13.48, 9.94)	0.873	n.i			
Q3	16.48 (−34.39, 67.36)			**19.67 (**−**26.17, 65.50)**		−2.32 (−13.07, 8.42)					
Q4	60.69 (8.51, 112.87)			**72.02 (26.06, 117.98)**		0.99 (−10.03, 12.02)					
*Contextual characteristics*											
Season (reference: May to August)											
September to November	11.35 (−33.42, 56.13)	0.617		n.i		1.59 (−7.53, 10.72)	0.730	n.i			
Mean percentage of greenspace (reference: Bottom quartile = lowest)											
2^nd^ quartile	−14.11 (−65.45, 37.22)	0.648		n.i		−5.93 (−16.44, 4.57)	0.139	−**4.39 (**−**15.21, 6.44)**	**0.036**		
3^rd^ quartile	−31.33 (−83.10, 20.44)					−11.80 (−22.40, −1.20)		−**13.69 (**−**24.77,** −**2.61)**			
4^th^ quartile (highest)	−6.24 (−56.04, 43.56)					−6.11 (−16.30, 4.09)		−**9.63 (**−**20.98, 1.71)**			
Urban Rural Status (reference: urban)											
Rural	18.37 (−18.37, 55.13)	0.325		n.i		−1.88 (−9.51, 5.74)	0.626	n.i			
Adjusted R squared				0.29				0.10			

n.i. not included as not significant in univariate analysis, **–** not added into the model as not relevant. β (95% CI) p indicates that regression coefficients, 95% confidence intervals and p values are given.

Among women, seven of the potential explanatory variables were associated with *reported* time spent in MVPA (Table 4) and three remained significant in the multiple regression model: having access to a bicycle and a standing or manual occupation showed positive associations, whilst having more than one child was negatively associated with reported MVPA. Eleven potential explanatory variables were associated with *recorded* time spent in MVPA, of which three remained significant in the multiple regression model: having a manual or standing occupation were positively associated with MVPA, whilst having access to a car, or being overweight or obese were negatively associated. For example, women who were overweight or obese recorded 6.9 fewer daily minutes in MVPA than those of normal weight (95% CI −12.2 to −1.8, *p* = 0.009).

**Table 4:** Associations between individual, socio-economic and area-level characteristics and time spent in moderate and vigorous physical activity in women.

	Average daily reported time in MVPA				Average daily recorded time in MVPA					
	Univariate associations β(95%CI) p		Multiple regressionmodel β (95%CI) p		Univariate associations β(95%CI) p				Multiple regressionmodel β (95%CI) p	
*Socio-demographic characteristics*										
Age group (reference: Under 40 years)										
40–50	−4.14 (−30.05, 21.76)	0.316	6.56 (−20.27, 33.39)	0.835	1.37 (4.75, 7.49)		0.511	2.59 (−3.53, 8.70)		0.529
Over 50 years	−12.11 (−35.67, 11.45)		2.05 (−22.38, 26.49)		−2.03 (−7.60, 3.53)			1.99 (−3.94, 7.92)		
Adults in the household (reference: Single person)										
Two adults	−14.83 (−42.14, 12.48)	0.086	−20.25 (−47.20, 6.70)	0.057	−3.60 (−10.06, 2.86)	0.086		−2.85 (−9.41, 3.70)		0.124
Three adults or more	−27.72 (−59.40, 3.96)		−32.17 (−63.90, −0.44)		−6.55 (−14.05, 0.93)			−5.55 (−13.18, 2.09)		
Children in the household (reference: None)										
At least 1 child in the household	−19.60 (−44.88, 5.67)	0.128	−**23.15 (**−**49.86, 3.56)**	**0.089**	−0.94 (−6.93, 5.05)	0.758		n.i		
Access to a bicycle (reference: no)										
Yes	36.82 (11.28, 62.36)	0.005	**38.20 (12.53, 63.86)**	**0.004**	3.76 (−2.33, 9.86)	0.226		1.40 (−4.67, 7.47)		0.650
Educational qualifications (reference: less than degree)										
Degree	46.69 (21.04, 72.34)	0.001	**42.32 (16.46, 68.18)**	**0.001**	10.35 (4.28, 16.42)	0.001		**8.72 (2.57, 14.87)**		**0.006**
Work type (reference: sedentary)										
Standing/Manual	10.87 (−10.67, 32.41)	0.322	n.i		−1.45 (−6.58, 3.67)	0.576		n.i		
Housing tenure (reference: rents home)										
Owns home	3.56 (−21.43, 28.55)	0.779	n.i		0.45 (−5.46, 6.36)	0.881		n.i		
Car access (reference: none)										
At least 1 car in the household	−0.69 (−37.00, 35.61)	0.970	n.i		−8.38 (−16.93, 0.15)	0.054		−**9.83 (**−**18.95,** −**0.71)**		**0.035**
Index of multiple deprivation (reference: least deprived)										
2^nd^ quartile	−25.98 (−54.27, 2.31)	0.487	n.i		0.43 (−6.26, 7.12)	0.239		−0.94 (−7.78, 5.90)		0.346
3^rd^ quartile	−7.11 (−35.07, 20.84)				1.33 (−5.28, 7.94)			−0.05 (−6.69, 6.58)		
Most deprived	−16.63 (−45.68, 12.42)				−4.98 (−11.85, 1.89)			−3.90 (−10.80, 3.00)		
Average daily wear time (in minutes)	–		–		0.073 (0.048, 0.010)	0.001		**0.06 (0.03, 0.09)**		**0.001**
*Health characteristics*										
Long term illness (reference: no)										
Yes	−5.98 (−37.43, 25.46)	0.708	n.i		−2.45 (−9.90, 4.99)	0.517		n.i		
Weight status (reference: normal weight)										
Overweight/Obese	−23.41 (−44.33, −2.50)	0.028	−19.46 (−40.89, 1.97)	0.075	−10.18 (−15.15, 5.20)	0.001		−**6.99 (**−**12.18,** −**1.79)**		**0.009**
Physical health summary score (reference: Q1)										
Q2	−2.07 (−31.77, 27.62)	0.995	n.i		1.69 (−5.27, 8.65)	0.021		1.22 (−5.81, 8.25)		0.100
Q3	10.80 (−17.61, 39.22)				7.52 (0.86, 14.18)			4.16 (−2.61, 10.93)		
Q4	−3.91 (−31.84, 24.02)				6.38 (−0.17, 12.92)			4.92 (−1.68, 11.51)		
Mental health summary score (reference: Q1)										
Q2	8.22 (−19.25, 35.68)	0.076	2.57 (−24.15, 29.29)	0.086	2.05 (−4.45, 8.56)	0.054		−0.04 (−6.49, 6.41)		0.078
Q3	1.83 (−26.89, 30.54)		1.35 (−27.25, 29.94)		5.59 (−1.21, 12.38)			4.37 (−2.51, 11.25)		
Q4	30.85 (1.62, 60.08)		28.14 (−0.60, 56.88)		5.85 (−1.07, 12.77)			4.93 (−1.82, 11.68)		
Contextual characteristics										
Season (reference: May to August)										
September to November	−20.15 (−43.03, 2.73)	0.084	−13.32 (−35.99, 9.36)	0.249	−3.81 (−9.22, 1.60)	0.166		−1.08 (−6.48, 4.32)		0.693
Mean percentage of greenspace (reference: Bottom quartile = lowest)										
2^nd^ quartile	11.80 (−16.91, 40.50)	0.332	n.i		−6.89 (−13.61, −0.18)	0.167		−4.13 (−10.89, 2.64)		0.102
3^rd^ quartile	8.87 (−20.46, 38.20)				0.47 (−6.39, 7.33)			1.41 (−5.35, 8.16)		
4^th^ quartile (highest)	16.50 (−12.92, 45.91)				2.33 (−4.56, 9.21)			4.18 (−2.88, 11.24)		
Urban Rural Status (reference: urban)										
Rural	9.81 (−10.63, 30.26)	0.346	n.i		2.35 (−2.48, 7.19)	0.339		n.i		
Adjusted R squared			0.07					0.13		

n.i. not included as not significant in univariate analysis, **–** not added into the model as not relevant β 95% CI indicates that regression coefficients, 95% confidence intervals and p values are given.

Although some of the associations between the potential explanatory variables and MVPA were not statistically significant, there was some evidence that the directions of associations tended to be consistent across both measures. For example, overweight or obese men both reported and recorded more minutes in MVPA per day on average. Women who were older, who had access to a bicycle and who reported higher mental health scores both reported and recorded more minutes of MVPA, and those who were overweight or obese and who completed questionnaires in the autumn engaged in fewer minutes of MVPA according to both measures.

## Discussion

### Principal Findings

In this sample of working age adults, few explanatory variables were associated with MVPA and few were similarly associated with both reported and recorded time spent in MVPA. The directions of associations between explanatory variables and reported and recorded MVPA tended to be more consistent in women than men.

### Strengths and Limitations

Causal associations cannot be inferred from these cross-sectional analyses. Our measures of reported and recorded MVPA were neither synchronous nor of matching duration: recorded MVPA was assessed over seven days, whereas reported MVPA was based on a four week recall period. However, it is unlikely that levels of MVPA differed significantly between the two measurement periods. Hip-worn accelerometers are known to underestimate cycling activity and arm movement and are not worn during waterborne activities such as swimming. [8] While more accurate methods exist for assessing physical activity energy expenditure, such as combined heart rate and movement sensors, [29] accelerometers were used in this study because they were easier to administer in a large postal study. While our classification of recorded MVPA used published cut points, [23] the optimal choice of cut points remains subject to debate. [30] Using different cut points in different studies may lead to different activities being classified at different intensities and can therefore make comparisons between studies difficult or, sometimes, inappropriate. However, we suggest that the different patterns of correlates observed in this study are unlikely to be sensitive to the precise cut point chosen.

More women than men participated in this study. This may explain the smaller and less consistent associations observed in men, which may reflect lower power to detect associations. Our sample also contains a higher proportion of participants educated to degree level and a smaller proportion of obese adults than the general population of Cambridgeshire [31], reflecting the focus of the study on healthy working adults. Our sample was also very active and relatively affluent, and therefore the correlates we identified here may not be generalisable to other populations. Despite these limitations, these initial analyses exploring the correlates of both *reported* and *recorded* time spent in MVPA in a relatively large sample of healthy working adults are important, given the relative lack of research in this area.

### Comparisons with Previous Literature

Our findings that the correlates of MVPA differed according to the physical activity measure used and that our models explained a larger proportion of the variance in men’s reported MVPA than in women’s are consistent with one of the few previous studies in this area. [10] In instances where the same correlates of physical activity were investigated, the direction of effect was often consistent across the two physical activity measures. For example, those authors observed a positive association between ‘blue collar’ occupations and physical activity; since blue collar occupations are less likely to be sedentary than professional or managerial occupations, their finding is consistent with our observation of a positive association between having a standing or manual occupation and MVPA. [10] As in that study, we also found a positive association between mental health summary score and physical activity. Given the consistent direction of effects, these explanatory variables may warrant further investigation in intervention studies; for example, it may be that interventions should be tailored for different subgroups of the population, or that those individuals who are less likely to be active should be identified and targeted in interventions to promote physical activity [32].

There were also some differences between the correlates observed in our study and those reported in the study by Cleland et al. [10] Firstly, we observed a positive association between occupation type and recorded physical activity in women, whereas they found no such association. This may reflect the different devices (pedometers versus accelerometers) and summary measures (‘steps/week’ versus ‘minutes of physical activity/week’ and ‘time spent in MVPA’) used for recorded physical activity in the two studies. Although both accelerometers and pedometers underestimate certain types of activities, pedometers do not capture the intensity of activity as accelerometers do. Secondly, those authors [10] reported a positive association between urban residence and reported physical activity in men, whereas we found no such association. Whilst the apparent contrast could reflect our relatively small sample of men, it could also represent true differences in the correlates of behaviour if these study samples engaged in different physical activities. In our sample, incidental activities such as walking and cycling were relatively common. In other population groups, organised physical activities requiring specialist facilities may be more commonly undertaken. Since these facilities are often located in urban areas, living in close proximity to such facilities may be positively associated with engagement in those activities. [33] Finally, the study by Cleland et al. [10] used a sample of young adults across Australia, whereas our study sample comprised healthy workers aged between 22 and 68 living in both urban and rural areas surrounding Cambridge.

### Trade-offs between Specificity and Practicality of Measurement

The comparison of the correlates of reported and recorded MVPA is highly dependent on the measures used. Despite using two validated measures of physical activity, [19], [22] we found some differences in the correlates of behaviour which probably reflect the relative strengths and weakness of the methods employed. For example, we found that access to a bicycle was negatively associated with recorded MVPA, but positively associated with reported MVPA. This is likely to reflect underascertainment by accelerometry of cycling, a behaviour which was particularly prevalent in men in our sample (69% of men and 56% of women reported cycling at least once in the last four weeks). This gives further strength to the argument that future research should use the appropriate device to capture the behaviours of interest; [34] while pedometers or accelerometers are likely to capture walking sufficiently, for example, other devices may be required to capture a wider range of physical activity behaviours. Given the limitations of any single method, one future direction of enquiry may be to investigate the potential to combine the two measures in analyses of the correlates of MVPA to balance their strengths and weaknesses. This may require different statistical techniques in analysis, such as the use of structural equation modelling [35] in which a latent variable representing ‘true’ physical activity would be estimated from direct measures of ‘reported’ and ‘recorded’ activity using a measurement model and the correlates of the latent physical activity variable subsequently modelled.

Furthermore, activities classified as being of ‘moderate-to-vigorous’ intensity cover a wide range including cycling, swimming and team sports – activities that are undertaken in a variety of settings and may be differentially related to individual, socio-demographic, health and contextual characteristics. With the collection of more context-specific behaviour data, perhaps using combinations of reported and recorded measures, analyses may be more likely to accurately capture the broader correlates of behaviour. This could be achieved by questioning participants more closely about where activity is undertaken or by using Global Positioning System (GPS) devices in combination with objective assessments of physical activity to identify where activities are undertaken [36].

### Conclusions

We found that the correlates of time spent in MVPA appeared to differ in both men and women according to the measures of physical activity being used. Researchers should recognise that the use of one particular objective measure of behaviour may influence the correlates which are observed. Given the limitations of both self-reported and objective measures of physical activity, researchers may wish to consider the use of both types of measures and investigate ways of combining these to gain a more complete view of the correlates of physical activity behaviour. Further research is required to assess the correlates of physical activity measured using different methods to clarify the differential associations observed here.

## References

[1] Bauman A, Bull F, Chey T, Craig C, Ainsworth B, et al (2009) The International Prevalence Study on Physical Activity: results from 20 countries. International Journal of Behavioral Nutrition and Physical Activity 31: 21. 10.1186/1479-5868-6-21 PMC2674408 19335883

[2] United Nations General Assembly (2011) Political declaration of the high-level meeting of the general assembly on the prevention and control of non-communicable diseases.

[3] Trost SG, Owen N, Bauman A, Sallis J, Brown W (2002) Correlates of adults’ participation in physical activity: review and update. Medicine & Science in Sports & Exercise 34: 1996–2001. 12471307 10.1097/00005768-200212000-00020

[4] Owen N, Humpel N, Leslie E, Bauman A, Sallis JF (2004) Understanding environmental influences on walking; Review and research agenda. American Journal of Preventive Medicine 27: 67–76. 15212778 10.1016/j.amepre.2004.03.006

[5] Sallis JF, Owen N (2002) editors (2002) Ecological models of health behavior.

[6] Duncan MJ, Spence J, Mummery K (2005) Perceived environmnet and physical activity:a meta-analysis of selected environmental characteristics. International Journal of Behavioral Nutrition and Physical Activity 2. 10.1186/1479-5868-2-11 PMC1236952 16138933

[7] Wendel-Vos W, Droomers M, Kremers S, Brug J, van Lenthe F (2007) Potential environmental determinants of physical activity in adults: a systematic review. Obesity Reviews 8: 425–440. 17716300 10.1111/j.1467-789X.2007.00370.x

[8] Janz KF (2006) Physical activity in epidemiology: moving from questionnaire to objective measurement. British Journal of Sports Medicine 40: 191–192. 16505072 10.1136/bjsm.2005.023036 PMC2492010

[9] Shephard RJ (2003) Limits to the measurement of habitual physical activity by questionnaires. British Journal of Sports Medicine 37: 197–206. 12782543 10.1136/bjsm.37.3.197 PMC1724653

[10] Cleland VJ, Schmidt MD, Salmon J, Dwyer T, Venn A (2011) Correlates of pedometer-measured and self-reported physical activity among young Australian adults. Journal of Science and Medicine in Sport 14: 496–503. 21622024 10.1016/j.jsams.2011.04.006

[11] Prince S, Adamo K, Hamel M, Hardt J, Gorber S, et al (2008) A comparison of direct versus self-report measures for assessing physical activity in adults: a systematic review. International Journal of Behavioral Nutrition and Physical Activity 5: 56. 18990237 10.1186/1479-5868-5-56 PMC2588639

[12] De Greef K, Van Dyck D, Deforche B, De Bourdeaudhuij I (2011) Physical environmental correlates of self-reported and objectively assessed physical activity in Belgian type 2 diabetes patients. Health & Social Care in the Community 19: 178–188. 20880106 10.1111/j.1365-2524.2010.00958.x

[13] Van Dyck D, Cardon G, Deforche B, Giles-Corti B, Sallis J, et al (2011) Environmental and psychosocial correlates of accelerometer-assessed and self-reported physical activity in Belgian adults. International Journal of Behavioral Medicine 18: 235–245. 21038103 10.1007/s12529-010-9127-4

[14] World Health Organization (2010) Global Recommendations on Physical Activity for Health. Geneva, Switzerland: World Health Orgnsation. 26180873

[15] Department of Health (2011) Start active Stay active A report on physical activity for health from the four home countries’ Chief Medical Officers. London: Department of Health.

[16] Hill JO, Wyatt HR (2005) Role of physical activity in preventing and treating obesity. Journal of Applied Physiology 99: 765–770. 16020440 10.1152/japplphysiol.00137.2005

[17] Ogilvie D, Giles-Corti B, Hooper P, Yang L, Bull F (2010) Methods for researching the physical activity impacts of ‘natural experiments’ in modifying the built environment. Journal of Physical Activity and Health 7: S341–S343.

[18] Ogilvie D, Griffin S, Jones A, Mackett R, Guell C, et al (2010) Commuting and health in Cambridge: a study of a ‘natural experiment’ in the provision of new transport infrastructure. BMC Public Health 10: 703. 21080928 10.1186/1471-2458-10-703 PMC2999608

[19] Besson H, Brage S, Jakes RW, Ekelund U, Wareham NJ (2010) Estimating physical activity energy expenditure, sedentary time, and physical activity intensity by self-report in adults. American Journal of Clinical Nutrition 91: 106–114. 19889820 10.3945/ajcn.2009.28432

[20] Ainsworth B, Haskell W, Whitt M, Irwin M, Swartz A, et al (2000) Compendium of physical activities: an update of activity codes and MET intensities. Medicine & Science in Sports & Exercise 32: S498–504. 10993420 10.1097/00005768-200009001-00009

[21] Hendelman D, Miller K, Baggett C, Debold E, Freedson P (2000) Validity of accelerometry for the assessment of moderate intensity physical activity in the field. Medicine & Science in Sports & Exercise 32: S442–S449. 10993413 10.1097/00005768-200009001-00002

[22] Plasqui G, Westerterp KR (2007) Physical Activity Assessment With Accelerometers: An Evaluation Against Doubly Labeled Water. Obesity 15: 2371–2379. 17925461 10.1038/oby.2007.281

[23] Freedson P, Melanson E, Sirard J (1998) Calibration of the Computer Science and Applications, Inc. accelerometer. Medicine & Science in Sports & Exercise 30: 777–781. 9588623 10.1097/00005768-199805000-00021

[24] World Health Organisation (2000) Obesity: preventing and managing the global epidemic. Report of a WHO consultation. Geneva: World Health Organisation. 1–253 p. 11234459

[25] Ware J, Kosinski M, Dewey J, Gandek B (2001) How to score and interpret singleitem health status measures: a manual for users of the SF-8 (TM) Health Survey. Lincoln, RI.

[26] Bibby P, Shepherd J (2004) Developing a New Classification of Urban and Rural Areas for Policy Purposes – the Methodology. London: Office for National Statistics. http://www.statistics.gov.uk/geography/downloads/Methodology_Report.pdf (accessed 3 Nov 2006).

[27] Department for Communities and Local Government (2005) Generalised Land Use Database Statistics for England 2005. London.

[28] Hosmer D, Lemeshow S (1989) Model-building strategies and methods for logistic regression. Applied Regression. New York: Wiley. 82–134.

[29] Brage S, Brage N, Franks PW, Ekelund U, Wareham NJ (2005) Reliability and validity of the combined heart rate and movement sensor Actiheart. Eur J Clin Nutr 59: 561–570. 15714212 10.1038/sj.ejcn.1602118

[30] Guinhouya CB, Hubert H, Soubrier S, Vilhelm C, Lemdani M, et al (2006) Moderate-to-vigorous physical activity among children: discrepancies in accelerometry-based cut-off points. Obesity 14: 774–777. 16855185 10.1038/oby.2006.89

[31] Office of National Statistics. Neighbourhood Statistics. Last accessed: 13.01.12. Available at. http://www.neighbourhood.statistics.gov.uk/dissemination/LeadHome.do.

[32] Ogilvie D, Foster CE, Rothnie H, Cavill N, Hamilton V, et al (2007) Interventions to promote walking: systematic review. BMJ 334: 1204. 17540909 10.1136/bmj.39198.722720.BE PMC1889976

[33] Humpel N, Owen N, Leslie E (2002) Environmental factors associated with adult’s participation in physical activity: A review. American Journal of Preventive Medicine 22: 188–199. 11897464 10.1016/s0749-3797(01)00426-3

[34] Tudor-Locke C, Ainsworth B, Thompson R, Matthews C (2002) Comparison of pedometer and accelerometer measures of free-living physical activity. Medicine & Science in Sports & Exercise 34: 2045–2051. 12471314 10.1097/00005768-200212000-00027

[35] Ishii K, Shibata A, Oka K (2010) Environmental, psychological, and social influences on physical activity among Japanese adults: structural equation modeling analysis. International Journal of Behavioral Nutrition and Physical Activity 7: 61. 20684794 10.1186/1479-5868-7-61 PMC2927494

[36] Krenn PJ, Titze S, Oja P, Jones A, Ogilvie D (2011) Use of Global Positioning Systems to Study Physical Activity and the Environment: A Systematic Review. American Journal of Preventive Medicine 41: 508–515. 22011423 10.1016/j.amepre.2011.06.046 PMC3821057

